# Magnetic microrheometry of tumor-relevant stiffness levels and probabilistic quantification of viscoelasticity differences inside 3D cell culture matrices

**DOI:** 10.1371/journal.pone.0282511

**Published:** 2023-03-22

**Authors:** Arttu J. Lehtonen, Ossi Arasalo, Linda Srbova, Maria Heilala, Juho Pokki

**Affiliations:** 1 Department of Electrical Engineering and Automation, Aalto University, Espoo, Finland; 2 Department of Applied Physics, Aalto University, Espoo, Finland; University of South Carolina, UNITED STATES

## Abstract

The progression of breast cancer involves cancer-cell invasions of extracellular matrices. To investigate the progression, 3D cell cultures are widely used along with different types of matrices. Currently, the matrices are often characterized using parallel-plate rheometry for matrix viscoelasticity, or liquid-like viscous and stiffness-related elastic characteristics. The characterization reveals averaged information and sample-to-sample variation, yet, it neglects internal heterogeneity within matrices, experienced by cancer cells in 3D culture. Techniques using optical tweezers and magnetic microrheometry have measured heterogeneity in viscoelasticity in 3D culture. However, there is a lack of probabilistic heterogeneity quantification and cell-size-relevant, microscale-viscoelasticity measurements at breast-tumor tissue stiffness up to ≃10 kPa in Young’s modulus. Here, we have advanced methods, for the purpose, which use a magnetic microrheometer that applies forces on magnetic spheres within matrices, and detects the spheres displacements. We present probabilistic heterogeneity quantification using microscale-viscoelasticity measurements in 3D culture matrices at breast-tumor-relevant stiffness levels. Bayesian multilevel modeling was employed to distinguish heterogeneity in viscoelasticity from the effects of experimental design and measurement errors. We report about the heterogeneity of breast-tumor-relevant agarose, GrowDex, GrowDex–collagen and fibrin matrices. The degree of heterogeneity differs for stiffness, and phase angle (i.e. ratio between viscous and elastic characteristics). Concerning stiffness, agarose and GrowDex show the lowest and highest heterogeneity, respectively. Concerning phase angle, fibrin and GrowDex–collagen present the lowest and the highest heterogeneity, respectively. While this heterogeneity information involves softer matrices, probed by ≃30 *μ*m magnetic spheres, we employ larger ≃100 *μ*m spheres to increase magnetic forces and acquire a sufficient displacement signal-to-noise ratio in stiffer matrices. Thus, we show pointwise microscale viscoelasticity measurements within agarose matrices up to Young’s moduli of 10 kPa. These results establish methods that combine magnetic microrheometry and Bayesian multilevel modeling for enhanced heterogeneity analysis within 3D culture matrices.

## Introduction

Changes in mechanical properties of human tissues relate to vital body functions [1–4], and the progression of diseases [5–8], including cancer [9–11]. In breast cancer, the cancer cells are surrounded by 3D extracellular matrix, and the matrix’s macromolecular organization is associated with mechanical properties that mediate invasion [11–15] and other critical behaviors [15–17] of cancer cells. The matrix characteristics have been taken into account in the recent 3D cell and tissue culturing methods that enable mimicking the accurate cancer-cell phenotype [15–18], protein expression [17], and critical biological pathways [18]. Yet, this 3D culturing, unlike conventional 2D culturing, typically requires the use of relevant scaffold matrices, which mimic the extracellular matrix within breast tumor tissues.

A variety of matrices [19–21] is used in 3D cultures to account for the mechanical [19] and biochemical properties within breast tumor tissues [22–26]. These matrices are often studied for mechanical properties using rheometers, revealing viscoelasticity of the matrix, or its (liquid-like) viscous and (stiffness-related) elastic characteristics, which have been found to mediate invasive cancer-cell migration [11, 14]. Rheometer measurements provide averaged values of viscoelastic properties, accompanied by information of the properties’ sample-to-sample variation [11, 18, 27]. Further, recent research shows that the matrix viscoelasticity not only involves sample-to-sample variation, but each of the matrix samples may exhibit internal variation that is referred to as heterogeneity in viscoelasticity [25]. Due to the heterogeneity, viscoelastic properties in multiple 3D-culture matrices vary spatially [25, 28–31]. Each cell senses the stiffness of its environment at the microscale [16, 32] as well as other viscoelasticity-related properties [11], and responds to those mechanical stimuli. The heterogeneity of 3D-culture matrices in the context of microscale viscoelasticity is yet to be comprehensively quantified.

Measuring the heterogeneity in viscoelasticity within 3D-culture matrices has mainly been carried out using two microrheological methods, optical tweezers [25] and magnetic microrheometers [33]. Optical tweezers measure at one individual location within a matrix at the time, with optional location-specific calibration for enhanced accuracy [34]. The measurements have mostly been carried out at the sample surface proximity (i.e. experimental depths are, typically, 10–50 *μ*m [25, 35], and exceptionally, up to 500 *μ*m [34]). Magnetic microrheometers enable simultaneous measurements of multiple locations [33], and the measurements can be performed within the depth on the order of millimeters [36]. However, magnetic microrheometers cannot currently perform cell-size-scale (1–100 *μ*m) viscoelasticity measurements within 3D-culture matrices that have an elevated stiffness level, a Young’s modulus up to ≃10 kPa [9], as in breast tumor tissues.

**Fig 1:**
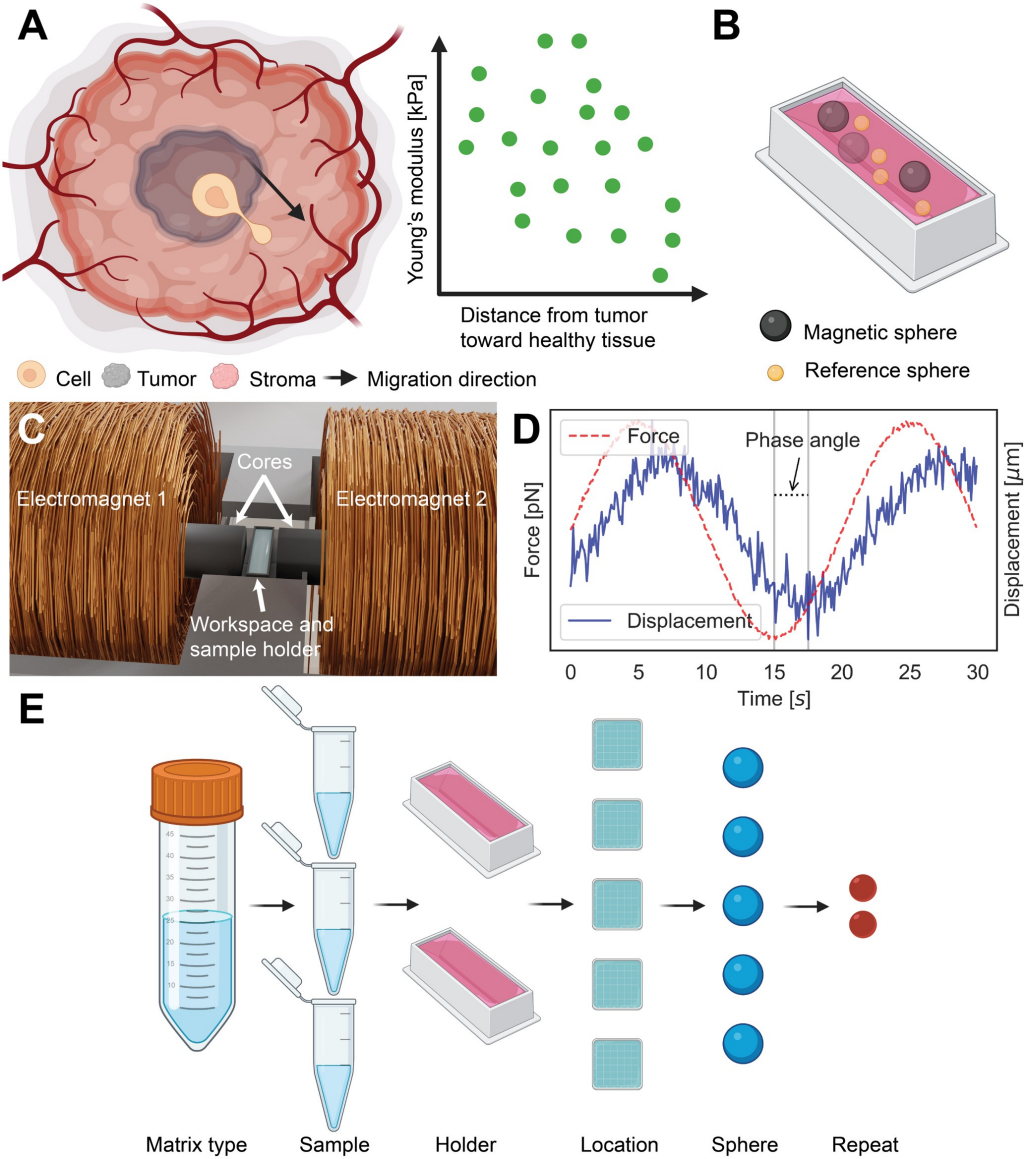
Microrheometry technique and experimental design for quantifying spatially differing microscale viscoelasticity. **A**: Breast-cancer tumor is surrounded by the stroma consisting of extracellular matrix. The cancer cells migrate out from the tumor, invade the stroma and further tissues during breast-cancer progression. In comparison to healthy tissues, the tumor tissues have often an increased Young’s modulus, and other viscoelastic properties also are altered [44]. **B**: Illustration shows a 3D culture matrix (in red) prepared into a sample holder. Two types of spheres, magnetic and reference spheres, have been introduced in the matrix during its preparation, enabling microscale viscoelasticity measurements using the microrheometer. **C**: The microrheometer system has two aligned electromagnets with cobalt–iron cores. The sample holders were placed into the working space between the cores. The electromagnets were driven by varied currents to exert forces on the magnetic spheres. **D**: Illustration of the oscillatory forces that were applied on a magnetic sphere. As a response, the magnetic sphere displaces in respect to reference, provided by reference spheres. The absolute shear modulus was derived using the amplitudes of the force and the displacement, while the phase angle was used as it is (indicated by the black vertical line). **E**: The sample preparation was carried out as in the multilevel hierarchy. For each matrix type, three samples were prepared, which were aliquoted into two sample holders per sample. Then, measurements were performed for 3–5 locations (field of view) within each holder. Two repetitive measuremens were provided. (Created with Biorender).

Here, we have developed magnetic-microrheometry methods, based upon [31, 33, 37], for measurements of microscale viscoelastic properties within 3D-culture matrices that have a range of Young’s moduli (E) relevant to breast-tumor tissue, from 100 Pa to 10 kPa (Fig 1A). The brief operating principle of the used magnetic microrheometer involves exertion of controlled forces onto ≃30 *μ*m or ≃100 *μ*m diameter magnetic spheres, and detection of the sphere-displacement responses. Initially, we calibrated the instrument using silicone oil. Then, we comprehensively quantified heterogeneity in viscoelasticity for the breast tumor tissue-relevant 3D-culture matrices that contain biologically inert agarose [18, 38] and GrowDex [18, 39], as well as biologically active GrowDex–collagen [40–42] and fibrin [43]. The heterogeneity quantification involved the use of a Bayesian hierarchical model incorporating the effects of experimental design to enable heterogeneity comparisons of the matrices. Finally, we probed the microscale viscoelasticity inside agarose matrices, providing Young’s moduli up to E = 10 kPa. The results establish methods that combine advanced magnetic microrheometry and the Bayesian hierarchical model for an enhanced heterogeneity analysis of spatially varying viscoelastic properties within 3D-culture matrices.

## Materials and methods

We quantified the heterogeneity in microscale viscoelasticity based on measurement spheres within breast tumor tissue (Fig 1A) relevant 3D-culture matrices (Fig 1B), and the use of the magnetic microrheometer (Fig 1C and 1D). Bayesian multilevel modeling was applied for the obtained microrheometry data to account for the experimental design (Fig 1E) and extract relevant heterogeneity metrics at stiffness levels up to a shear modulus (G) of 0.4 kPa. The ≃30 *μ*m magnetic spheres used in the heterogeneity quantification experience insufficient forces to induce detectable displacements at higher stiffness levels (i.e. G > 0.4 kPa). Therefore, larger spheres, the ≃100 *μ*m magnetic spheres, were used to increase the magnetic volume-dependent forces, responsible for the displacements. Thus, measurements were demonstrated at the breast-cancer tumor tissue-relevant stiffness levels (i.e. up to shear moduli (G) of 3.5 kPa, or Young’s moduli (E) of 10–11 kPa). The microscale viscoelasticity measurements were validated using conventional parallel-plate rheometry. The related codes are available on https://github.com/ArttuD/microrheology.

### Measurement spheres

We incorporated measurement spheres within the matrices to quantify the matrix heterogeneity. We used two sphere types, magnetic and non-magnetic spheres, which had the final volume fractions of 0.06% and 0.03%, respectively. The magnetic spheres were composed of iron oxide and poly(lactic acid), and had mean nominal diameters of either 30 *μ*m (Micromod GmbH, 12–00-304), or 100 *μ*m (Micromod GmbH, 12–00-105), which we refer to as ‘the 30 *μ*m magnetic spheres’ and ‘the 100 *μ*m magnetic spheres’, respectively. Uncoated magnetic spheres were used for all matrices, except for agarose, which required coating with NH_2_ groups to achieve homogeneous dispersion. Non-magnetic spheres with a nominal mean diameter of 6.0 *μ*m were used in all matrices to provide a reference location for the magnetic probes in order to remove mounting-related and environmental vibrations. The suspension containing either the 30-*μ*m- or the 100-*μ*m-diameter magnetic spheres, accompanied by the non-magnetic spheres, is referred to as ‘sphere suspension’.

### 3D culture matrices

We used four types of 3D culture matrices—agarose, GrowDex (nanofibrillar cellulose), double-network GrowDex–collagen, and fibrin—that contained the sphere suspension. For the microrheometry measurements, the matrices were prepared in 25 x 4.5 x 3.3 mm^3^ polymethylmethacrylate holders that had a microscopy cover glass glued underneath.

For agarose matrices, the stock solution was prepared by dispersing ultrapure low melting point agarose (Invitrogen, 16520050) in Milli-Q water. The solution was placed in a water bath at 80°C until the powder dissolved. The warm solution was immediately mixed with the required amounts of the sphere suspension to achieve the desired final concentration of agarose: 0.5%, 0.9%, 1.0%, 1.1%, 1.25%, and 1.35%. Before the sample solution cooled down, it was pipetted into the sample holders. The sample was then allowed to gel at room temperature for 50 minutes. The heterogeneity quantification employed the 30 *μ*m magnetic spheres for the 0.5% agarose, whereas the pointwise microscale viscoelasticity measurements used the 100 *μ*m magnetic spheres for the rest of the agarose concentrations.

GrowDex matrices were prepared using a 1.5% GrowDex product (UPM Kymmene Oyj, 100103002) by diluting the product to a concentration of 1.25%. The required volumes of Milli-Q water and non-magnetic spheres were transferred into one mixing syringe, and a female-to-female Luer lock connector was attached. Subsequently, the 1.25% GrowDex solution was aliquoted into another mixing syringe, and the 30 *μ*m magnetic spheres were added directly into the GrowDex solution, to prevent adhesion of the spheres to the plastic parts of the syringe. The two syringes were then connected, and the contents were mixed 50 times, by alternately pushing the syringe plungers, to homogenize the sample. The contents were finally aliquoted into sample holders and let sit for 30 minutes at the room temperature before the measurements.

Double-network GrowDex–collagen matrices were prepared using a 1.5% GrowDex product and a rat-tail collagen type I product (Corning, 354236) that were diluted to a 0.45% solution and a 2.0 mg/mL concentration, respectively. The pH was maintained at ≃8. The required volumes of DMEM/F12 medium, non-magnetic sphere suspension, GrowDex, and 1.045% NaOH were transferred into the first mixing syringe and a Luer lock connector was attached. Subsequently, the collagen type I was aliquoted into the second mixing syringe and the 30 *μ*m magnetic sphere suspension was added directly into the solution. Then, the syringes were connected, and the two constituents were mixed 50 times for homogenization. The solution was promptly aliquoted into sample holders and allowed then for polymerization at 37°C for 50 minutes.

To prepare fibrin matrices, chilled solutions of human plasma fibrinogen (Sigma-Aldrich, F3879) in PBS–Tris buffer (0.5xPBS, 507 mM Tris, pH 8.8) and human plasma thrombin (Sigma-Aldrich, T6884) in 0.024% bovine serum albumin (Biowest, 9048–46-8) were combined using a dual-barrel syringe. The unpolymerized gel solution was maintained in a cooling block and mixed with the 30 *μ*m sphere suspension to reach the final fibrinogen content of 30 mg/mL and 12 NIH U/mL thrombin. The solution was immediately transferred to the sample holders, and allowed for polymerization for 30 min at room temperature.

### Scanning electron microscopy (SEM)

A SEM (Zeiss Sigma VP FEG) was used to characterize the morphology of the magnetic spheres and the surrounding matrices (i.e. the 30 *μ*m spheres in a 0.5% agarose matrix, and the 100 *μ*m spheres in a 1.0% agarose matrix). These samples were prepared for the SEM as followed. First, the samples were gradually dehydrated with a series of ethanol concentrations (10, 30, 60, 80, 90 and 100%), each for 1 day at room temperature. Subsequently, they were dried using the critical point method, to minimize the morphological alterations commonly caused by lyophilization. Loose magnetic spheres were collected with magnet. These dried samples were mounted on stubs using carbon tape, sputter coated with 5 nm of Pt/Pd, and finally imaged using the SEM.

### Microrheometry for microscale viscoelasticity measurements

Microscale viscoelastic properties of the 3D culture matrices were measured using the matrix-embedded magnetic spheres and a previously developed magnetic microrheometer [33]. The instrument was modified to use a Basler 3.2 MP microscope camera on a Zeiss Axiovert 200M microscope. The microrheometer had electromagnets with an outer and inner diameters of 80 mm and 6 mm, respectively, with lengths of 47 mm, which were positioned to generate a cylindrically symmetric magnetic field along the axis perpendicular to the electromagnet cross section. The two electromagnets had cobalt–iron cores (Vacuumschmelze, Vacoflux 50), with diameters of 6 mm, and lengths of 65 mm. The inner blunt sides of the cores were placed 7 mm from each other to provide a space for the sample holder.

The measurements were based on the exertion of magnetic forces by the microrheometer (i.e. micromanipulator type 1 [33]). The microrheometer exerted forces on the magnetic spheres within the matrices, via the generation of magnetic-field gradients and constant magnetic fields by the electromagnets, as described in [33, 45]. Briefly, the field gradients tune the forces exerted on the magnetic spheres, of which magnetization is set by the fields [33, 45]. These force-generating field gradients and the fields can be separately adjusted using superpositioned currents (Eqs 1 and 2), fed to the electromagnets [33, 45].

Previous works by Pokki et al. [33, 45] provide a detailed characterization of the homogeneity of magnetic fields and magnetic-field gradients for the microrheometer system type. In this work, we simulated the microrheometer system according to the ref. [46] to model the force-altering magnetic-field gradients during the sinusoidal current sequence. Fig S1A and S1B in S1 File these simulation results for magnetic-field gradients (∇*B*_*z*_). Cylindrical coordinates were used and a rotational symmetry was assumed (i.e. z axis and x axis are parallel and perpendicular to the electromagnet core’s long axis, respectively). The simulations were performed to the sinusoidal current sequence used in the experiments (i.e. sinusoidal amplitude based on *i*_grad_ = 1.25 A, and a constant *i*_field_ = 0.75 A maintained the magnetic-sphere magnetization; see Eqs 1 and 2). The workspace is indicated with red vertical lines. The simulation’s workspace is relevant to the field of view of the experiments (i.e. 560 *μ*m × 420 *μ*m). Further, the matrix types are elastically dominated materials (i.e. phase angle (*ϕ*) is less than 20°; see Results). Therefore, the relevant homogeneity of the field gradients (∇*B*_*z*_) is for the maximal/minimal field gradients generating maximal/minimal forces on the magnetic spheres (Fig S1A–S1B in S1 File; time points of 5 s and 15 s). Thus, these field gradients correspond to the peak-to-peak values of the sinusoidal displacement responses. The homogeneity of the force-generating field gradients (∇*B*_*z*_) around the center of workspace is ± 12%. Within this workspace, Pokki et al. [33] report symmetrical magnetic fields and a high degree of homogeneity of the fields.

This force generation by the two electromagnets was controlled by two distinct currents, driven by linear amplifiers using bipolar 40 V power supplies (GW, SPD-3606). The current-controlled forces were applied on the magnetic spheres. A Labview program and a DAQ card (National Instruments, PCle-6341) were used to set the two time-varying currents fed to the electromagnets 1–2 to generate the forces and induce magnetic-sphere displacements (Fig 1C).

The currents supplied to both electromagnet coils (*i*_coil 1_ and *i*_coil 2_) used an equal offset (*i*_offset_) values of 0.75 A. Each offset value was superpositioned by a sinusoidally varying current (*i*_grad_) with an amplitude of 1.25 A and a frequency of *f* = 0.05 Hz:

$$\begin{array}{cc} & i_{\text{coil}\;\text{1}}=i_{\text{offset}}+i_{\text{grad}} \end{array}$$ (1)

$$\begin{array}{cc} & i_{\text{coil}\;\text{2}}=i_{\text{offset}}-i_{\text{grad}} \end{array}$$ (2)

The magnetic spheres were tracked in respect to the non-magnetic reference spheres, using recorded videos. The tracked displacements (*p*_spheres_) were fit to the following equation:

$$\begin{array}{c} p_{\text{spheres}}={\hat{p}}_{\text{spheres}}\;\text{sin}\;\left(2\pi ft-\phi \right), \end{array}$$ (3)

where ${\hat{p}}_{\text{spheres}}$ is the amplitude of the displacement signal, *f* is the frequency, *ϕ* is the phase angle between the applied force and the observed displacement, and *t* is time.

The absolute shear modulus (|*G*|) was calculated based on the fitted ${\hat{p}}_{\text{sphere}}$ values:

$$\begin{array}{c} \left|G\right|=\frac{{\hat{F}}_{\text{volumetric}}V_{\text{sphere}}}{3\pi D_{\text{sphere}}{\hat{p}}_{\text{sphere}}}, \end{array}$$ (4)

where ${\hat{F}}_{\text{volumetric}}$ is the volumetric force constant derived during the calibration, *V*_sphere_ is each magnetic sphere’s volume, and *D*_sphere_ is the diameter of each magnetic sphere. NB: The volumetric force constant denotes for ${\hat{F}}_{\text{volumetric}}=\frac{{\hat{F}}_{\text{sphere}}}{V_{\text{sphere}}}$, where ${\hat{F}}_{\text{sphere}}$ is the magnetic-force amplitude exerted on each magnetic sphere.

To compare to breast-cancer tissue stiffness [9], the calculated |*G*| values of agarose matrices were converted to Young’s modulus (E) values assuming that the Young’s modulus is equivalent to the absolute Young’s modulus (i.e. E = |E|):

$$\begin{array}{c} E=2(1+v)|G|, \end{array}$$ (5)

where *v* is the Poisson’s ratio of the agarose matrices (i.e. *v* = 0.37–0.50) [47, 48].

The microrheometer was initially calibrated in respect to the instrument-generated magnetic forces, and the phase angle. The calibration was realized by using dried magnetic and reference spheres that were resuspended in silicone oil with known viscous properties (i.e. dynamic viscosity of *μ* = 30 000 cSt; Sigma, 63148–62-9). The silicone oil, containing the dried spheres, was aliquoted into the sample holders.

During the calibration, we determined the working space, and quantified subsequently the average magnetic-force amplitude acting on the distinct-sized magnetic spheres within the workspace (i.e. the average volumetric force constant ${\hat{F}}_{\text{volumetric}}$). Briefly, the calibration protocol involved exerting the current-controlled, sinusoidal magnetic forces on the magnetic spheres in silicone oil, and tracking (details in S1 File) the magnetic-sphere displacements in respect to reference spheres, enabling derivation of the ${\hat{F}}_{\text{volumetric}}$. The volumetric force constant was calculated based on the Stokes’ drag force (i.e. $F_{\text{d}}=3\pi \mu D_{\text{sphere}}{\hat{v}}_{\text{sphere}}$, where *μ* is the dynamic viscosity of the silicone oil at room temperature, and ${\hat{v}}_{\text{sphere}}$ is the average fitted velocity amplitude of a magnetic sphere). Thus, the volumetric-force constant is:

$$\begin{array}{c} {\hat{F}}_{\text{volumetric}}=\frac{F_{\text{d}}}{V_{\text{sphere}}}=\frac{3\pi \mu D_{\text{sphere}}{\hat{v}}_{\text{sphere}}}{\frac{4}{3}\pi {\left(D_{\text{sphere}}/2\right)}^{3}}=18\mu \frac{{\hat{v}}_{\text{sphere}}}{{D_{\text{sphere}}}^{2}} \end{array}$$ (6)

This relation shows that—for a constant ${\hat{F}}_{\text{volumetric}}$—the velocity amplitude (${\hat{v}}_{\text{sphere}}$) of each magnetic sphere has a linear relation with the squared diameter of the sphere (${D_{\text{sphere}}}^{2}$), as well as with the squared sphere radius ($r_{\text{sphere}}^{2}=(\frac{D_{\text{sphere}}}{2}{)}^{2}$). Thus, instead of calculating ${\hat{F}}_{\text{volumetric}}$ separately for each magnetic sphere, we have estimated the slope (*β*) between the two variables ($\beta =\frac{{\hat{v}}_{\text{sphere}}}{{r_{\text{sphere}}}^{2}}$) to get a more robust estimate of the ${\hat{F}}_{\text{volumetric}}$ constant for calibration. Thus, we have fitted a simple linear regression model without an intercept. The model is as follows:

β∼N(0,5000), (7)

$$\sigma ∼\text{Inverse-Gamma}\left(0.5,1\right)\cdot 10^{-8},$$ (8)

$${\hat{v}}_{\text{sphere}}∼N(\beta r_{\text{sphere}}^{2},\sigma^{2}),$$ (9)

where *σ* is an unknown standard deviation. The model’s prior choices have been scaled to match the scale of the data. The model assumes a Gaussian likelihood. Given the model, the ${\hat{F}}_{\text{volumetric}}$ constant is simply the following: ${\hat{F}}_{\text{volumetric}}=\frac{9}{2}\beta \mu$. The goodness of fit has been assessed with a Bayesian *R*^2^ value [49]. In addition to this ${\hat{F}}_{\text{volumetric}}$ constant calculation, the distribution of the phase angles (*ϕ*) for each magnetic sphere was recorded to evaluate the measurements uncertainty.

In this work, the phase angle (*ϕ*) was used in the analysis instead of the conventional loss tangent (tan(*ϕ*)). For describing the ratio between the viscous and elastic characteristics, the phase angle was considered simpler, since it is also descriptive for purely viscous materials (*ϕ* = 90°)—such as the silicone oil—that have infinite loss-tangent values.

The experimental design followed the hierarchy specified in Fig 1E. For each matrix type, we prepared three samples, and each sample was split to two sample holders. For each sample holder, 3–5 locations containing 1–3 magnetic spheres were measured at room temperature. The samples that failed to polymerize normally (≃0 Pa) or contained the spheres mostly at the bottom of the sample holder were unused in the further analysis.

The measurements were carried out at the linear viscoelasticity regime, since the forces exerted by the microrheometer provide small displacements within the matrices, specifically, the 30 *μ*m and the 100 *μ*m magnetic spheres displace at the most hundreds of nanometers. By using these small forces/displacements, we avoid detection of nonlinearities in the matrix viscoelastic properties (e.g. for agarose [50]), develop a measurement protocol that would minimize distraction to cells due to sphere motion in 3D culture, and avoid unnecessary heating of the electromagnets applying the forces. According to Eq 4, the 100 *μ*m spheres are expected to measure ≃8 times larger |*G*| values in comparison to the 30 *μ*m spheres (i.e. $\frac{|G_{\text{100}}\;\mu \text{m}|}{|G_{\text{30}}\;\mu \text{m}|}≃8$; derivation in S1 File). The measured viscoelastic properties can be assigned to specific points within the matrix, whereas—in an ideal homogeneous material—such values are constant at each point/location. The most of the used 3D culture matrices are known to exhibit heterogeneous or spatially varying viscoelastic properties [25, 28–30].

### Bayesian multilevel modeling of heterogeneity in viscoelasticity

**Fig 2:**
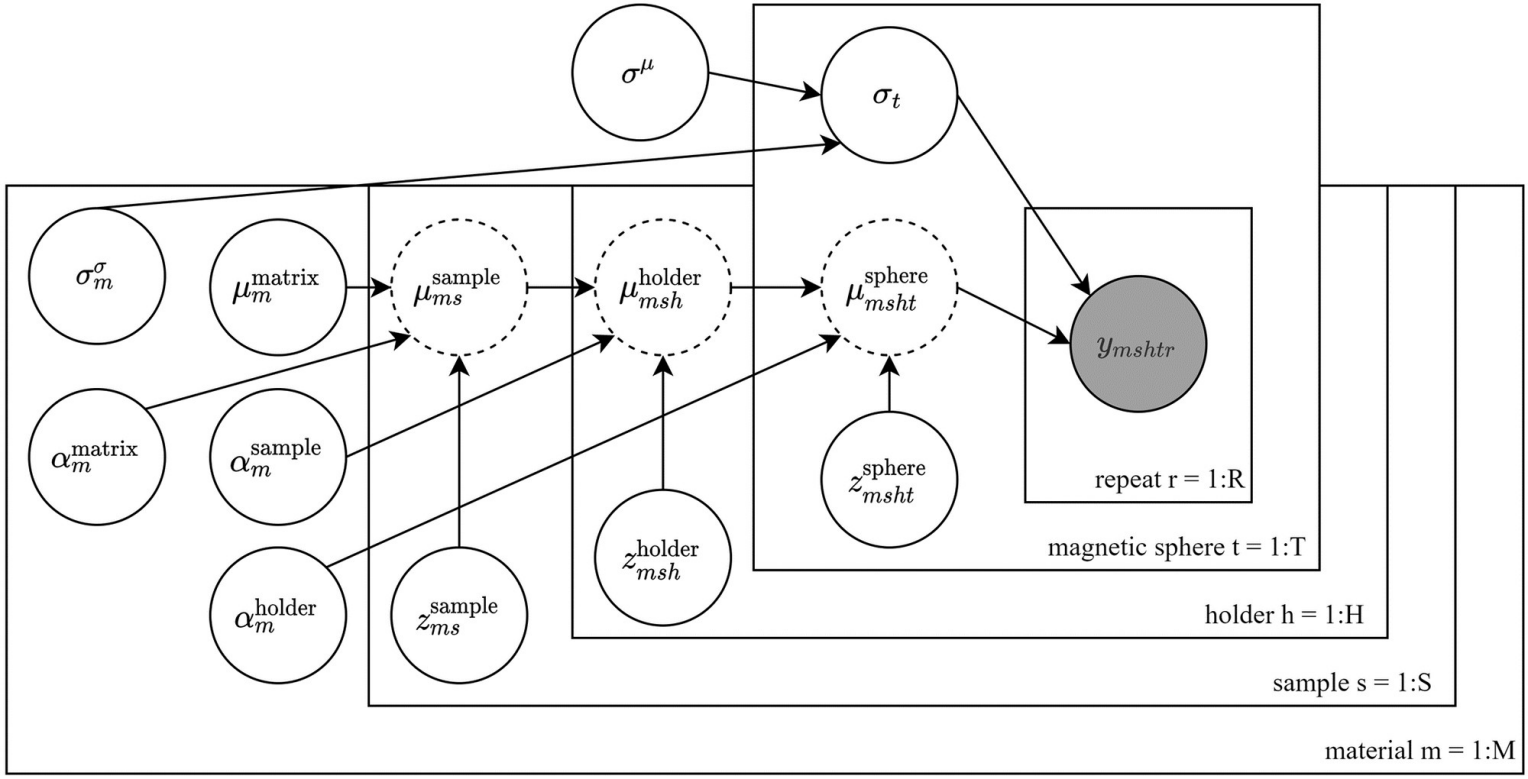
Heterogeneity model definition for quantifying spatial variation of viscoelastic parameters in 3D culture matrices. The mean value of each magnetic sphere is estimated as a sum of multiple level effects. There are *m* different matrix materials, *h* holders, *s* samples, *h* magnetic beads, and *r* repeats. Different 3D matrix material types are independent of each other, apart from sharing a common mean noise term. Partial pooling is used for each level of hierarchy to improve robustness. Specific level effects are denoted by a parameter vector *μ*, where the superscript is the level, and the subscript is the parameter corresponding the given observation (m, h, s, or t). The scale of the effect is provided by *α* with analogous super/subscripts. Non-centered parametrization is used for the level effects that are denoted using dotted lines (Eq 14). This model is run separately for estimating the absolute shear modulus (|*G*|) and the phase angle (*ϕ*) (denoted by *y* with subscripts). The gray color indicates observations (i.e. either |*G*|, or *ϕ*). The used data has been z-score normalized.

Conventional metrics of defining heterogeneity, such as coefficient of variation, have challenges in interpretability and they may include combined information of both spatial variation and experimental uncertainty [51–53]. We captured the 3D culture matrices’ heterogeneity of microscale viscoelasticity, using a Bayesian multilevel model, separating the spatial variation from the known experimental uncertainty. The experimental design structure (Fig 1E) can be incorporated into this model to robustly estimate the heterogeneity. The Bayesian paradigm was chosen due to its strength in quantifying uncertainty, which is especially important since the data sizes were relatively small (i.e. 3 samples per matrix type). The 3D culture matrices’ heterogeneity was assumed to manifest itself at the sample-holder level, therefore, the heterogeneity is defined as the magnitude of the spatial variation in viscoelastic properties within a sample holder. The used model is summarized in Fig 2 and explained in detail in the following paragraphs. The model variables are listed below:

$$\mu_{m}^{matrix},z_{ms}^{sample},z_{msh}^{holder},z_{msht}^{sphere}∼N(0,1)\;\text{Level}\;\text{effect}\;\text{priors}$$ (10)

$$\alpha_{m}^{matrix},\alpha_{m}^{sample},\alpha_{m}^{holder}∼HalfNormal(0,1)\;\text{Level}\;\text{effect}\;\text{priors}$$ (11)

$$\sigma_{m}^{\sigma },\sigma^{\mu }∼HalfNormal(0,1)\;\text{Noise}\;\text{priors}$$ (12)

$$\sigma_{t}∼InverseGamma(\sigma^{\mu },\sigma_{m}^{\sigma })\;\text{Measurement}\;\text{noise}$$ (13)

$$\mu_{ms}^{sample}=\mu_{m}^{matrix}+\alpha_{m}^{matrix}z_{ms}^{sample}\;\text{Sample}\;\text{effect}$$ (14)

$$\mu_{msh}^{holder}=\mu_{ms}^{sample}+\alpha_{m}^{sample}z_{msh}^{holder}\;\text{Holder}\;\text{effect}$$ (15)

$$\mu_{msht}^{sphere}=\mu_{msh}^{holder}+\alpha_{m}^{holder}z_{msht}^{sphere}\;\text{Sphere}\;\text{effect}$$ (16)

$$y_{mshtr}∼N(\mu_{msht}^{sphere},\sigma_{t})\;\text{Likelihood}$$ (17)

We quantified the heterogeneity in viscoelasticity using this multilevel model that has the level effect priors as in Eqs 10 and 11. Specifically, we extracted the 3D culture matrix type-specific $\alpha_{m}^{\text{holder}}$ value for both |*G*| and *ϕ* data (Eq 11). The matrix type-specific values were used to provide the heterogeneity information within each holder, accounting for the uncertainty (Eqs 12 and 13) and the experimental design (Eqs 14–16). The likelihood was chosen to be a Gaussian distribution (Eq 17).

This primary model was constructed as a three level, linear random intercept model with partial pooling at each level. The levels were chosen to account for the errors in experimental design. As demonstrated in Fig 1D, each matrix type condition was divided into samples (*s* = 1, …, *S*), which consisted of holders (*h* = 1, …, *H*), locations within holders, and finally, repeated measurements (*r* = 1, …, *R*) by individual magnetic spheres within each location (*t* = 1, …, *T*). The model estimated the viscoelastic properties based on the magnetic spheres, by computing the sum of the sample, sample holder, and sphere level information (Eqs 14–16). Each parameter is a vector and the subscript denotes the correct index for the *i*th observation. For instance, $\mu_{msht}^{sphere}$ means the magnetic sphere effect for the *m*th material/condition (e.g agarose), *s*th sample, *h*th holder and *t*th magnetic sphere within the described nested structure.

The location level was unaccounted in this primary model due to the following challenges in the results interpretability. If the location level had been used, the matrix heterogeneity would have most likely been an underestimate of the total spatial variation. Additionally, the parameter of interest would have been the location-dependent variation, which is problematic as the experimental protocol typically enabled 1–3 measurements of the 30 *μ*m magnetic spheres in a location. Further, the parameter describing the variation in viscoelasticity within a holder would irrelevantly represent the variation between locations.

The measurements included noise for the detected displacements of each magnetic sphere. Therefore, each measurement by a magnetic sphere was replicated at least two times to enhance accuracy. The measurement uncertainty differed between magnetic spheres, due to the errors in the particle tracking. Therefore, a separate noise term was introduced for each individual magnetic sphere. Similarly as the level estimates, this noise term *σ*_*t*_ was partially pooled to improve robustness. The mean noise level, indicated by *σ*^*μ*^, was shared over 3D-culture matrices as it is reasonable to consider the mean noise level to be invariable, because the same microrheometer system was used. On the other hand, the variation of the noise ($\sigma_{m}^{\sigma }$), is independent for each material, owing to random effects caused by the distinct properties of the matrices.

For numerical stability, the data (|*G*| and *ϕ*) was z-score normalized. The normalization’s standard deviation was the same for each individual 3D culture matrix to keep the comparison of the heterogeneity comparable. In contrast, the normalization used the mean values of each matrix. NB: The magnitudes of the 3D culture matrix properties were irrelevant in this heterogeneity quantification. Further, accompanied by this normalization, the level effects were reparameterized with non-centered parametrization, as explained in [54]. The model was written in Stan probabilistic programming language [55] and sampled with the default Hamiltonian Monte Carlo parameters provided by the Stan library.

The model performance was validated by confirming that the Markov Chain Monte Carlo (MCMC) algorithm had explored the parameter space fully and converged. An out-of-sample predictive performance was estimated with expected log pointwise predictive density (ELPD). This was done via a cross validation by leaving one magnetic sphere out at a time (details in S1 File). The model was also compared against other versions of the hierarchical model to validate the model choices as described below (Figs S2–S5 in S1 File for all model specifications).

Initially, the primary model (model 1, Fig 2) was compared against a model with an additional location level effect (model 2, Fig S2 in S1 File). The interpretation of the parameters changes in the model 2, where the variable $\alpha_{m}^{\text{holder}}$ (Eq 11) lacks itself to indicate the material heterogeneity but rather the variation of locations within each sample. NB: The additional location-level effect ($\alpha_{m}^{\text{location}}$) captures the variation in viscoelasticity based on the spheres within a location. On the other hand, the used model 2 captures the experimental-design effects in $\alpha_{m}^{\text{holder}}$.

Then, comparisons were performed against three further models (models 3–5, Figs S3–S5 in S1 File) that are expected to overestimate the heterogeneity as they are incapable of accounting for the effects of experimental design. The primary model (model 1) was compared against the model 3 with only a single level of hierarchy assuming no sample, holder or location level effects. For the model 4 (baseline), the primary model (model 1) was compared against the fully pooled model 4 where the material had a single mean value (of |*G*| or *ϕ*) and the standard deviation of the mean indicated the matrix heterogeneity. The importance of the pooled noise term (model 5) was also confirmed by removing it from the baseline model (model 4) as specified in Fig S4 in S1 File. Table S1 in S1 File provides further details on the model comparisons.

Additionally, the primary hierarchical model results were compared against measurements from parallel-plate rheometry. As discussed earlier, the conventional parallel-plate rheometry is unable to capture the samples’ internal spatial heterogeneity, but quantifies the sample-to-sample variation. Thus, we analyzed the microrheometry data by using the hierarchical model’s estimate of the sample-to-sample variation ($\alpha_{m}^{\text{sample}}$; Fig 2) for comparison to the parallel-plate rheometry data based on the conventional coefficient of variation (i.e. the ratio between standard deviation and mean).

### Parallel-plate rheometry for macroscale validation

A rheometer (Physica MCR 302 rheometer, Anton Paar), with a parallel plate of 25 mm in diameter, was used to validate the microrheometry results of the 3D culture matrices. The rheometry experiments were carried out as follows. A volume of 1 mL of a pre-gel matrix solution was deposited onto the bottom plate of the rheometer. The upper smooth plate of the rheometer was immediately lowered to the desired gap height of 1 mm. The excess sample was trimmed from the edges, and an oil enclosure was formed around the sample to prevent water evaporation. Time sweeps were performed at a strain amplitude of 1%, using a frequency of 0.05 Hz, at a temperature of 25°C. The absolute shear modulus and the phase angle were measured every 1 min throughout the gelation process, until the sample reached an equilibrium state or the measurements were stopped after 90 minutes.

This protocol had minor exceptions in the measurements of GrowDex–collagen and fibrin matrices. The GrowDex–collagen matrices were measured using two temperature intervals (at a frequency of 0.05 Hz). During the first interval lasting for 40 min, the temperature was set to 37°C to let collagen to polymerize. In the second interval, lasting from 40 min to 90 min, the temperature was set to 25°C. Further, the rheological properties of the fibrin matrices were measured using a similar instrument, AR2000 (TA Instruments), having a 20-mm-diameter parallel plate. A fibrin pre-gel was loaded between the plates at 4°C, and the parallel-plate geometry was covered with a solvent trap to prevent evaporation. The temperature was raised to 20°C and the fibrin gelation took place in 30 min. After the gelation, 30 min time sweep measurement was performed at a 1% strain amplitude and at a 0.16 Hz frequency. In comparison to microrheometry, the parallel-plate rheometry’s slightly higher frequency is expected to have a negligble effect, since the fibrin-matrix viscoelasticity is insensitive to frequency in the measurements at frequencies up to 1.0 Hz [28].

In addition, we investigated the effects of the magnetic and reference spheres on the rheology of agarose and GrowDex matrices. These matrices were prepared with and without the spheres, and measured using the parallel-plate rheometer (Fig S6 in S1 File). The samples with the spheres were prepared and measured as described before, but—for the samples without the spheres—the volume taken by the spheres was replaced with Milli-Q water.

## Results

The results include the microrheometer calibration and the quantification of heterogeneity in viscoelasticity for the different 3D culture matrix types. Further, the pointwise microscale viscoelasticity at tumor-relevant stiffness levels is revealed, via increased magnetic-force measurements, obtained by enlarging the magnetic-probe nominal diameter, from 30 *μ*m to 100 *μ*m.

### Calibration of the microrheological approach

**Fig 3:**
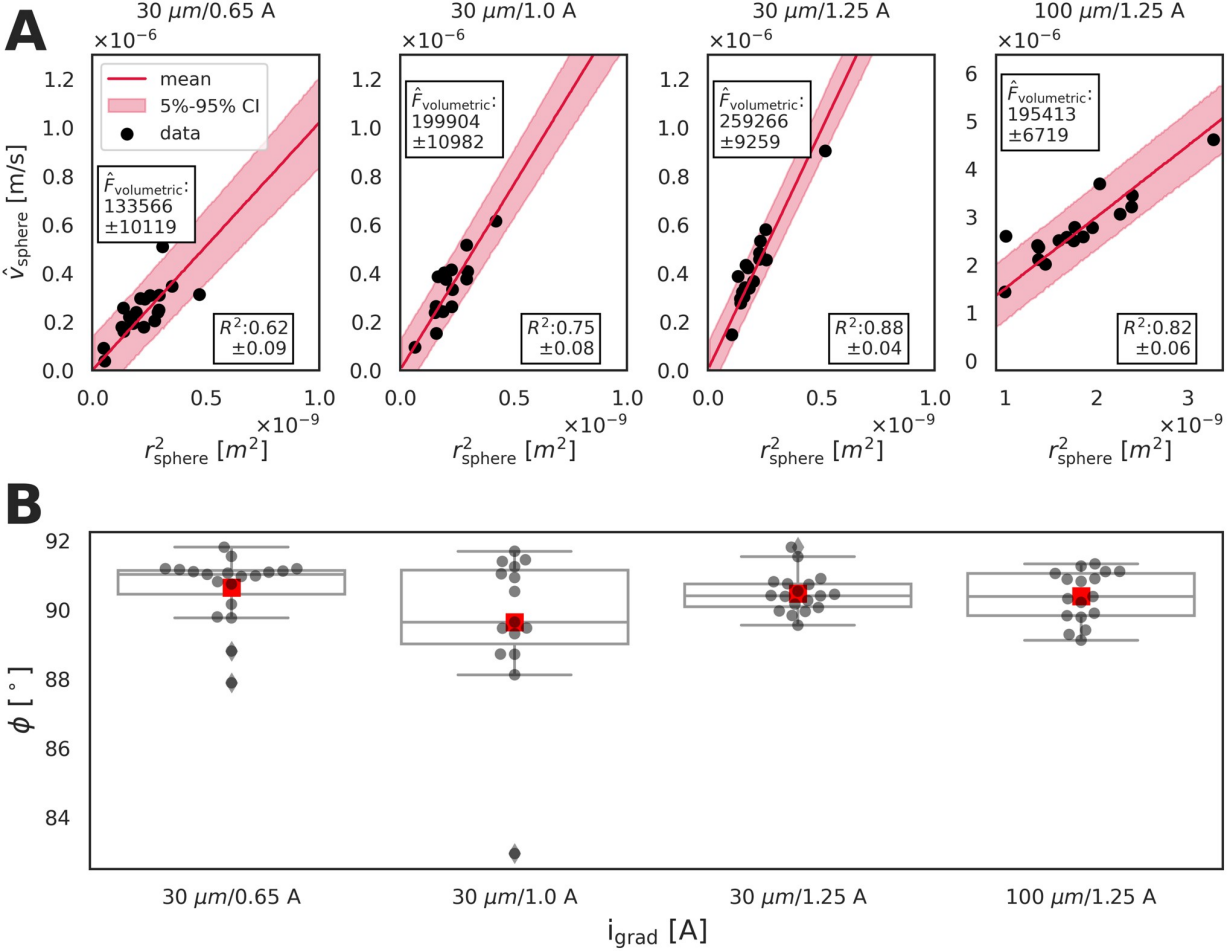
Microrheometer calibration based on volumetric force (${\hat{F}}_{\text{volumetric}}$) and phase angle (*ϕ*) as a function of varied currents (*i*_grad_). **A**: Relation between the squared sphere radius ($r_{\text{sphere}}^{2}$) and the average sphere velocity (${\hat{v}}_{\text{sphere}}$) is shown as black spheres. The *R*^2^ values show the goodness of fit of the linear curves [49]. The red shadowed areas indicate the credible intervals of the estimates. Increasing the value of i_grad_ enlarges the measured volumetric forces (i.e. steeper slope) exerted onto the magnetic spheres, as previously reported [45]. As the calibration for both the 30 and 100 *μ*m spheres, the ${\hat{F}}_{\text{volumetric}}$ constant values at *i*_grad_ = 1.25 A were calculated based on the linear fits (red line), and used in all further experiments. **B**: Relation between the phase angle (*ϕ*) and the current i_grad_ is presented. The measurements are scattered around the ideal value of 90° for purely viscous materials. The dark grey spheres show the *ϕ* values measured by individual magnetic spheres, while the red squares are the mean values of the spheres. Standardly, the plots’ box and whiskers indicate the lower/upper extremes, the 25% and the 75% percentiles, and the median. Each dataset indicates whether the calibration is for the 30 *μ*m or the 100 *μ*m spheres.

The microrheometer calibration involves the determination of the relationship between the electromagnet current i_grad_, and the force applied on the magnetic spheres within the silicone oil. We report on the use of force-adjusting, electromagnet currents *i*_grad_ = 0.65 A, *i*_grad_ = 1.00 A, and *i*_grad_ = 1.25 A, and measurements of the volumetric force (${\hat{F}}_{\text{volumetric}}$) (Fig 3A and Table 1) and the phase angle (*ϕ*) (Fig 3B). As the magnetic spheres have differences in radii that scales the magnetic volume, the volumetric forces exerted on the spheres have been quantified by accounting for both the varying radii and the volume (Eqs 6–9). Specifically, determining linear fits between the squared sphere radii and averaged velocity amplitude of the spheres enables extraction of the volumetric force (Fig 3A and Eq 7). The data follows the linear trend for all the datasets and the fits have an increasing certainty for the volumetric forces at elevated currents *i*_grad_. Further, Fig 3A shows that the current *i*_grad_, related to magnetic-field gradient, expectedly adjusts the volumetric force applied on the spheres. Table 1 shows the calibration of the ${\hat{F}}_{\text{volumetric}}$ constant values derived from the slopes of the linear fits for both the 30 *μ*m and 100 *μ*m magnetic spheres. Additional comparisons against another, less accurate estimate of the ${\hat{F}}_{\text{volumetric}}$ constant calculated as magnetic-sphere specific values is in Table S2 and Fig S7 in S1 File. Fig 3B shows phase-angle values that are distributed around the predicted value of 90° for purely viscous fluids, and the values are independent from *i*_grad_.

**Table 1:** Calibration in respect to volumetric force (${\hat{F}}_{\text{volumetric}}$). The ${\hat{F}}_{\text{volumetric}}$ constants are shown as mean and standard deviation values (*σ*).

sphere nominal diameter [*μ*m]	*i*_grad_	mean [*N*/m^3^]	*σ* [*N*/m^3^]
30	0.65	133566	10119
30	1.00	199904	10982
30	1.25	259266	9259
100	1.25	195413	6719

All further experiments are carried out at *i*_grad_ = 1.25 A, providing the highest force for maximal displacement signal-to-noise ratio. The mean ± standard deviation values of the volumetric force for the 30 *μ*m and 100 *μ*m magnetic spheres are ${\hat{F}}_{\text{volumetric},30}=2.59\cdot 10^{5}$ N/m^3^ ± 0.09 ⋅ 10^5^ N/m^3^ and ${\hat{F}}_{\text{volumetric},100}=1.95\cdot 10^{5}\pm 0.07\cdot 10^{5}$ N/m^3^, respectively. The volumetric-force uncertainty is described by the ratio between standard deviation and mean: 3.6% for the 30 *μ*m magnetic spheres, and 3.4% for the 100 *μ*m magnetic spheres (Table 1). The volumetric forces correspond to maximum forces of 12.8 nN for the 30 *μ*m spheres, and 154.2 nN for the 100 *μ*m spheres.

### Heterogeneity in microscale viscoelasticity for the 3D culture matrix types

Next, we quantified the heterogeneity of microscale viscoelastic properties inside four different 3D-culture matrix types: agarose, fibrin, GrowDex–collagen, and GrowDex (Fig S8 in S1 File). These matrices are at the softer range of breast-tumor-related tissue (i.e. the mean |*G*| ≃ 100–200 Pa). The full summarized mean viscoelasticity values (|*G*| and *ϕ*) of these different matrix types and their concentrations are shown in Table 2. Further, supplementary structural heterogeneity information about the used matrix types is available (i.e. agarose [50], fibrin [28, 56], and GrowDex [39]).

**Table 2:** Microrheometry for the viscoelasticity of the matrix types.

matrix type	|*G*| [Pa]		*ϕ* [°]	
	mean	standard deviation *σ*	mean	standard deviation *σ*
Agarose 0.5%	80.79	44.09	5.46	3.01
Agarose 0.9%	949.33	316.27	4.85	1.98
Agarose 1.0%	1433.95	372.60	6.34	2.59
Agarose 1.1%	2421.81	608.48	4.66	4.87
Agarose 1.25%	2412.16	703.11	8.80	8.46
Agarose 1.35%	3567.79	2101.59	8.74	6.34
Fibrin	165.09	112.87	5.55	2.50
GrowDex	229.50	166.96	12.36	4.78
GrowDex–collagen 0.45%/2.0 mg/mL	117.66	117.55	19.35	8.91

**Fig 4:**
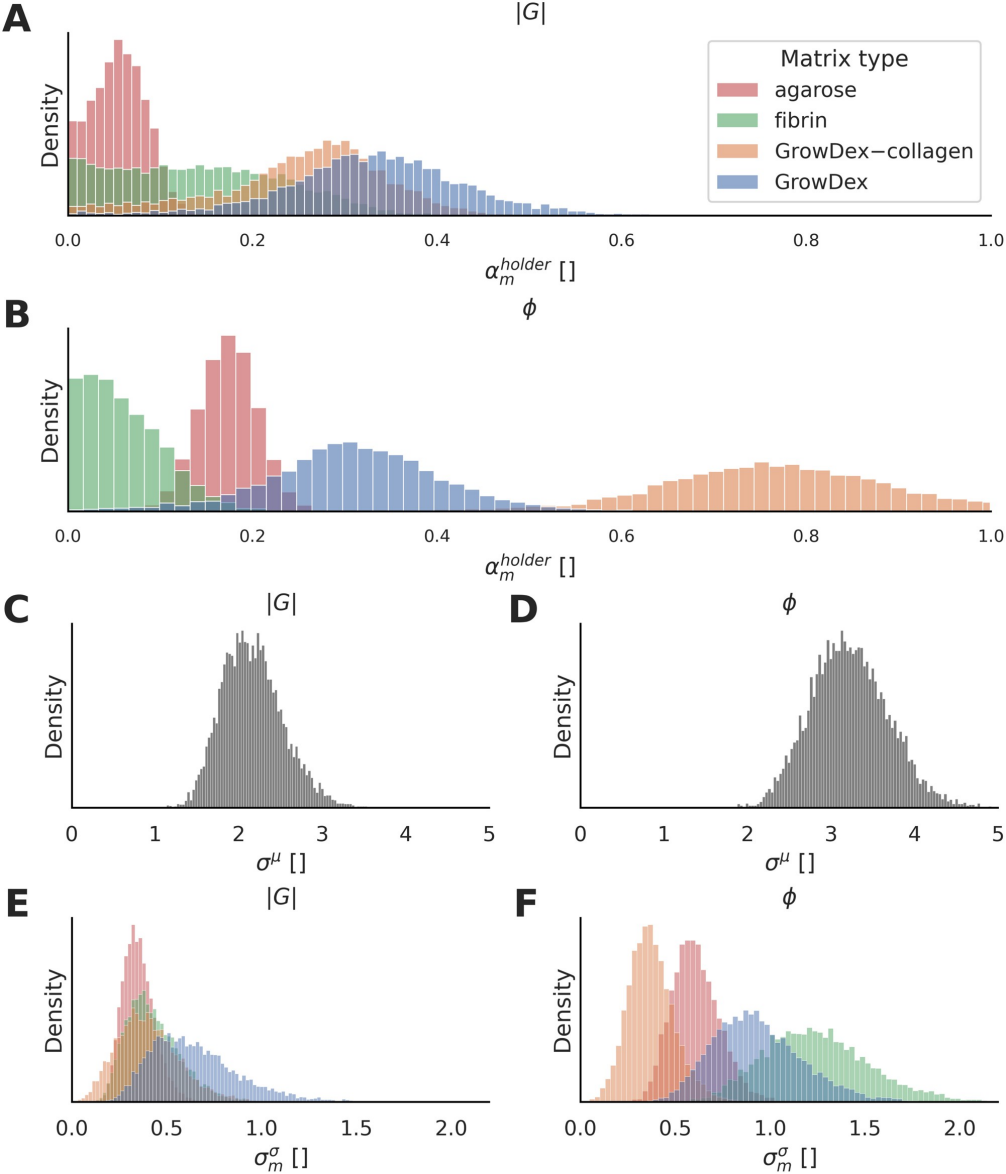
Multilevel Bayesian model enables distinguishing of the heterogeneity in matrix viscoelasticity from the effects of experimental design and measurement errors. **A–B**: Posterior distributions of absolute shear modulus (|*G*|) and phase angle (*ϕ*). For each matrix type, the distribution peak describes the mean of the heterogeneity estimate, while the distribution width indicates uncertainty. **C–D**: Estimated average measurement errors are shown for |*G*| and *ϕ*. **E–F**: Estimated error’s variability between individual magnetic spheres are shown for |*G*| and *ϕ*. The distribution peak describes the magnitude of the estimated error’s variability in each matrix type. For all plots, the X axes show the |*G*| data or the *ϕ* data that are z-score normalized (unitless), and the Y axes represent the probability density of the distributions (unitless).

We used the Bayesian multilevel model to quantify the heterogeneity of the absolute shear modulus (|*G*|) and the phase angle (*ϕ*) within each matrix type (Fig 4A and 4B). We report the posterior distributions of the heterogeneity parameter ($\alpha_{m}^{\text{holder}}$), indicating the spatial variability, for the viscoelastic properties (|*G*| and *ϕ*). The peak value and the width of the distributions describe the magnitude and uncertainty of the heterogeneity in the viscoelastic properties, respectively. The number of samples, sample holders and imaged locations that have passed the data post-processing pipeline are summarized in Table S3 in S1 File.

Concerning the absolute shear modulus (i.e. stiffness), agarose and GrowDex are the least and the most heterogeneous matrices based on the model (Fig 4A). The differences between fibrin, GrowDex and GrowDex–collagen remain uncertain. Regarding the phase angle, fibrin is the least heterogeneous and the heterogeneity increases in the following order: agarose, GrowDex and GrowDex–collagen (Fig 4B). Pairwise heterogeneity differences are further compared using probability values for absolute shear modulus and phase angle (Table S4 in S1 File).

Besides heterogeneity quantification, the model estimates the measurement error and the estimated error variation across the magnetic spheres used within each matrix type. The distribution in Fig 4C and 4D estimates the average measurement errors and Fig 4E and 4F show the variation of the estimated errors. This measurement-error estimation accounts for the fore-mentioned factors, as well as the uncertainty due to the relation between absolute shear modulus and displacement signal amplitude (Fig S9 in S1 File).

**Fig 5:**
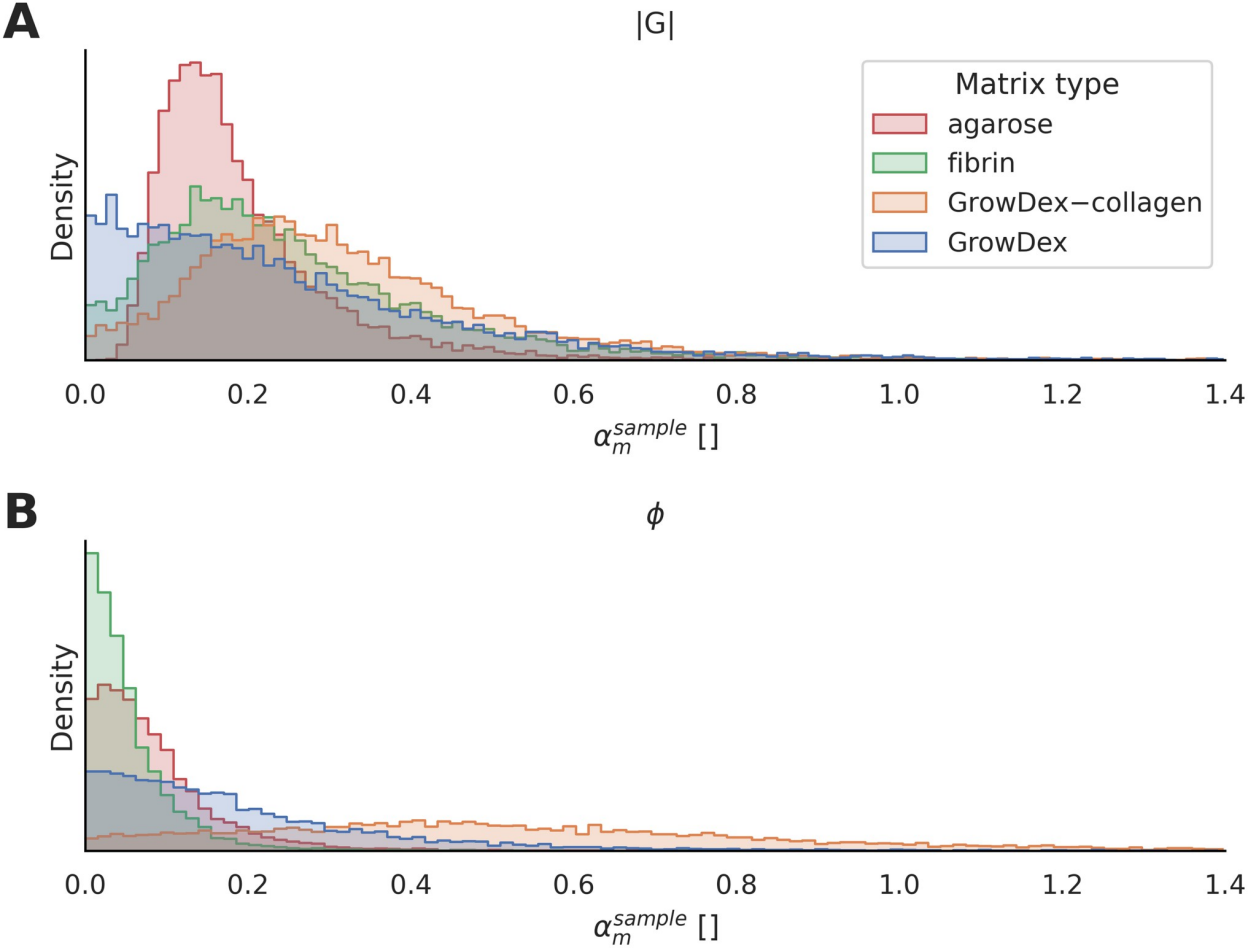
Sample-to-sample variation in microrheology experiments based on the hierarchical Bayesian model. The peak values indicate the level of heterogeneity for shear modulus (|*G*|) and phase angle (*ϕ*). The peak-value results suggest the same order of increasing heterogeneity between matrix types as parallel-plate experiments (Table 3), except for the phase-angle result of the fibrin matrix. Uncertainty is indicated by the overlap between the distributions.

To compare the correspondence between microrheometry and parallel-plate rheometry, we also quantified sample-to-sample variation for each of the matrix types. The microrheometry results using the Bayesian model are summarized in Fig 5, and the results from the parallel-plate experiments are shown in Table 3. The results suggest that both microrheometry and parallel-plate rheometry have the same order of increasing sample-to-sample variation for the matrix types, in respect to shear modulus (|*G*|). Correspondingly, the results are similar for phase angle (*ϕ*), except for fibrin, which has the smallest sample-to-sample variation in microrheology and the third smallest value (or the second highest value) in parallel-plate rheometry. Fig 5 shows the distributions behind the microrheometry results that have a high degree of overlap indicating uncertainty.

**Table 3:** Parallel-plate rheometry for comparing sample-to-sample variation. Sample-to-sample variation is quantified based on coefficient of variation that is the ratio between standard deviation (*σ*) and mean. The coefficients of variation for shear modulus (*c*_*v*,|*G*|_) and for phase angle (*c*_*v*,*ϕ*_) are shown.

matrix type	|*G*| [Pa]		*ϕ* °[]		*c*_*v*,|*G*|_	*c*_*v*,*ϕ*_
	mean	*σ*	mean	*σ*		
Agarose 0.5%	117.627	11.218	1.361	0.033	0.095	0.024
Fibrin	177.695	34.388	3.146	0.385	0.194	0.123
GrowDex	359.690	21.256	6.737	0.403	0.060	0.060
GrowDex–collagen 0.45%/2.0 mg/mL	75.526	42.367	9.710	2.173	0.561	0.224

### Pointwise microscale viscoelasticity measurements at tumor-relevant stiffness levels

**Fig 6:**
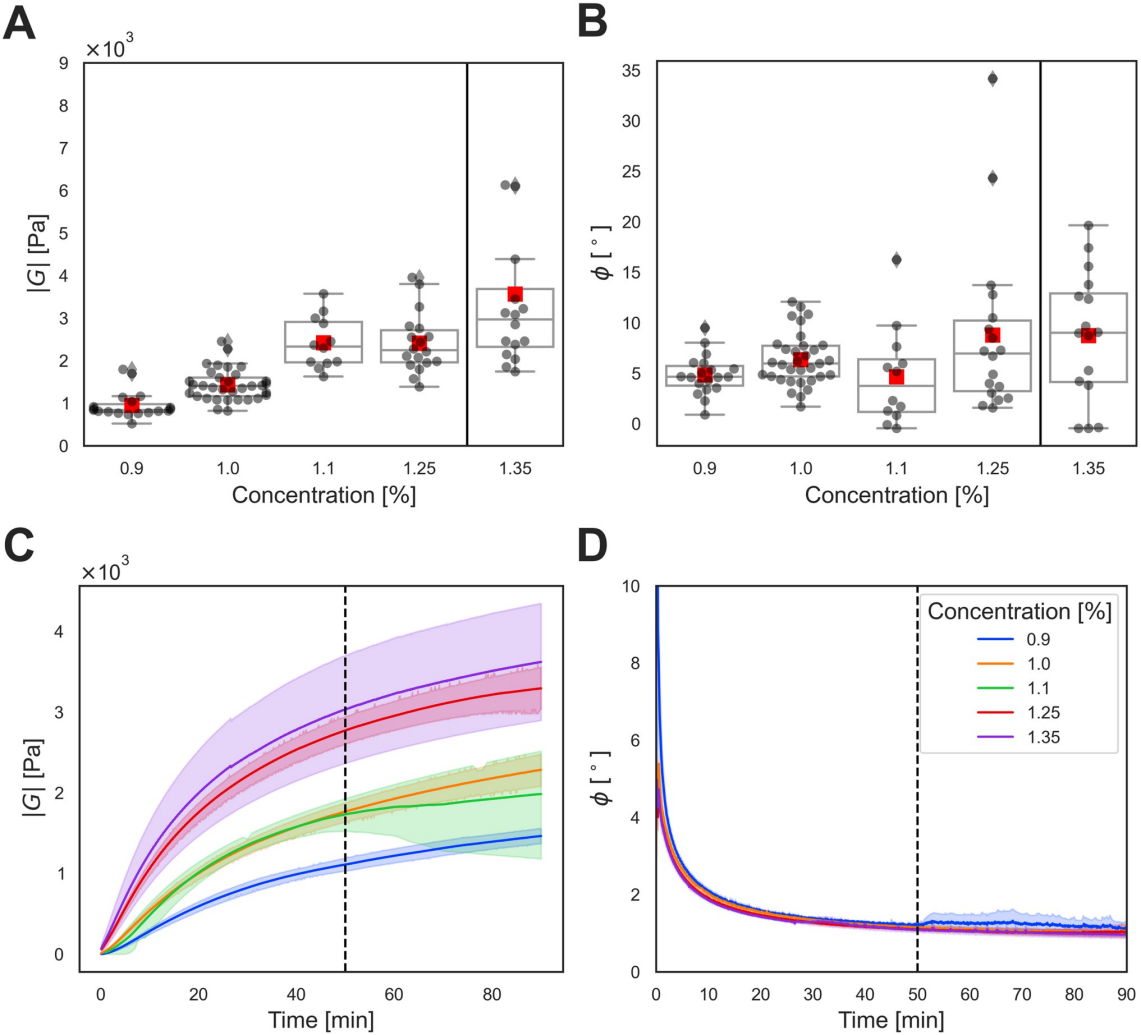
Pointwise microscale viscoelasticity measurements of breast tumor tissue-relevant stiffness levels. **A–B**: Absolute shear modulus (|*G*|) and phase angle (*ϕ*) values of agarose matrices with increasing concentration measured using the 100 *μ*m magnetic spheres and the magnetic microrheometer. Agarose matrices with a concentration up to 1.25% can be measured, whereas the signal-to-noise ratio significantly reduces for higher concentrations, including 1.35%, for which measurements are considered unsuccessful. Therefore, the 1.35% agarose condition is separated by a black vertical line from other measurements. The successful microrheometer measurements of |*G*| for the 1.25% agarose matrix with mean ± standard deviation values of 2.41 ± 0.70 kPa correspond to Young’s moduli (E) from 6.61 ± 1.93 kPa (*v* = 0.37) to 7.24 ± 2.11 kPa (*v* = 0.50). Multiple individual measurements of |*G*| exceed 3.5 kPa, denoting 10–11 kPa in Young’s modulus (*v* = 0.37–0.50). For both plots, the dark grey spheres show values measured by individual magnetic spheres, while the red squares are the mean values of the spheres. Standardly, the plots’ box and whiskers indicate the lower/upper extremes, the 25% and the 75% percentiles, and the median. **C–D**: Absolute shear modulus (|*G*|) and phase angle (*ϕ*) values of the agarose matrices at the used concentrations were validated using parallel-plate rheology. The dashed vertical line corresponds to a timepoint of 50 min after the start of the measurements, relevant to microrheological measurements. The curves with darker colors indicate the mean values of the data for each agarose concentration, while the lighter shaded-color regions indicate the lower 5% and the upper 95% percentiles for the data.

We demonstrate the ability of the microrheometer system to measure microscale viscoelasticity within 3D culture matrices at physiologically relevant stiffness levels (Fig 6A and Table 2). The prepared agarose matrices with concentrations from 0.9% to 1.25% have comparable Young’s moduli from 1 kPa to 10 kPa (Eq 5) as in a breast tumor tissue [9]. For the stiffest 1.35% agarose matrices, the analysis pipeline underperformed due a low displacement signal-to-noise ratio. The measurements determined the upper bound of stiffness for the used analysis pipeline and the microrheometer. Further, the phase-angle values of the agarose matrices with the varying concentration are shown in Fig 6B and Table 2. Parallel-plate rheometry is used to confirm the measured absolute shear modulus and phase angle values (Fig 6C and 6D). These microrheology measurements that report the microscale values within each sample are well distributed around the average value obtained with the macroscale parallel-plate rheometry, validating the measurements.

## Discussion

We have advanced methods [31, 33, 37] to quantify microscale differences in viscoelasticity within 3D culture matrices that have a stiffness (E) range relevant to breast-tumor tissue, from 100 Pa to 10 kPa. We specifically report on three pertinent areas: the microrheometer’s calibration/uncertainty, heterogeneity within different matrix types, and pointwise measurements at breast-tumor relevant stiffness levels.

We provide a microrheometer calibration procedure that is independent from the 3D culture matrix type. The calibration enables comparisons between the matrix types, since the calibration coefficients are independent of material properties. Moreover, the microrheometer neither requires any further location-specific calibration before each measurement, nor is limited to the vicinity of the sample surface. Forces exerted on the magnetic spheres are symmetric in the working space between the electromagnets. Further, the following factors impact the results uncertainty. We observed that the silicone oil-based calibration involved no disturbing local flows generated by the forces exerted on the 30 *μ*m magnetic spheres. Yet, the 100 *μm* magnetic spheres experienced local flows that provided dragging of some reference spheres. Despite of this, the calibration using the 100 *μ*m magnetic spheres provides microrheometry results that are consistent with macroscale parallel-plate rheometry. Further, as theoretically expected in Eq 4, the absolute shear modulus has a squared dependence on the magnetic sphere diameter. Therefore, the diameter estimation is crucial for accurate results. Further, it is unfeasible to exclude all the spheres with morphological defects from the analysis, because brightfield microscopy provides 2D projection of the spheres. The SEM images reveal that a portion of spheres posses deviation from sphericity and this deviation is a key error source in the experiments (Fig S10 in S1 File). Additionally, the choice of the magnetic-sphere coating is important, since magnetic spheres have distinct chemicophysical interactions with the matrix material which depend on the matrix type and coating of the magnetic spheres [45]. The smaller 30 *μ*m magnetic spheres, with an appropriate coating for each matrix type, provide a sufficient sphere density within the microscope’s field of view for the heterogeneity quantification. Further, we observed a discrepancy of volumetric-force constants between the 30 and 100 *μ*m magnetic spheres at *i*_grad_ = 1.25 A (Table 1). Based on the datasheet, they are made of the same material. The 25% difference in volumetric force could be due to a deviation of magnetic-material densities between these sphere types.

A heterogeneity quantification approach to estimate microscale spatial differences in viscoelasticity inside each 3D culture matrix type has been developed. Quantifying the viscoelastic cues is crucial to deepen the understanding of individual cell behavior and its relation to matrix viscoelasticity, extensively studied by [14, 32]. We used the Bayesian multilevel model to provide an interpretable heterogeneity quantification of the viscoelastic properties within four matrix types. Based on the model, the agarose matrices are the least heterogeneous in stiffness (|*G*|) compared to the GrowDex, GrowDex–collagen and fibrin matrices. Particularly, the specific order of the latter three matrices is uncertain, suggesting similarity of heterogeneity in stiffness. Further, the degree of heterogeneity for the phase-angle results differed to the ones for the stiffness results. The heterogeneity in phase angle increases in the following order: fibrin, agarose, GrowDex, and GrowDex–collagen. These quantifications report on the heterogeneity differences between the matrix types that have variation in stiffness, thus, the quantifications are unintended to account for stiffness-related heterogeneity differences. The amount of data for each matrix type is expected to impact the heterogeneity uncertainty, observed as the distribution width (Fig 4A and 4B). For example, the agarose matrices with the largest dataset (Table S3 in S1 File) have the least uncertainty based on the narrowest distributions. Further, the model enables us to assess further the uncertainty in the estimated heterogeneity due to the microrheometer-based measurements. The estimated measurement errors on average are consistent between the different matrix types (Fig 4C and 4D). The model quantifies and accounts for the variation of the estimated errors between the individual magnetic probes, specific to each matrix type (Fig 4E and 4F). Further, the quantified displacements of magnetic spheres decrease for increased microscale stiffness, which reduces the displacement signal-to-noise ratio, enlarging the uncertainty of the estimated heterogeneity. Therefore, the width of the distributions is expected to increase for the stiffer matrix types (e.g. GrowDex) (Fig 4A and 4B). Unexpectedly, the estimates of the measurement error in stiffness are highly overlapping between matrix types indicating consistency of errors regardless of the matrix type (Fig 4E), and also the distribution widths are mainly similar (Fig 4A). Expectedly, the estimated error variability in phase angle is higher for the stiffest matrix type (GrowDex) and differs between the matrix types (Fig 4F), having variable distributions widths (Fig 4B).

While the direct comparison of the heterogeneity results by the microrheometry with parallel-plate rheometry is unfeasible, these microrheometry results can be indirectly compared to parallel-plate rheometry via the underlying sample-to-sample variation. For shear modulus, the order of increasing sample-to-sample variation of the matrix types is the same between these techniques (Fig 5). For phase angle, the order of the sample-to-sample variation is also the same, expect for fibrin matrix, having a large overlap of the distribution with other matrices, indicating an elevated uncertainty. These findings suggests consistency between the techniques, although all the distributions behind the microrheometry results have significant overlap related to uncertainty (Fig 5).

Pointwise measurements at stiffer breast tumor-tissue relevant stiffness levels were carried out. For the purpose, we replaced the 30 *μ*m magnetic spheres, probing softer matrices, with the larger 100 *μ*m magnetic spheres that provide sufficient magnetic forces for probing stiffer matrices. Since these larger magnetic spheres enable measuring only one sphere within the microscope’s field of view, less heterogeneity data can be collected at the larger size scales [57]. Therefore, we present pointwise microscale measurements without quantification of heterogeneity. Specifically, the highest measured stiffness levels up to E = 10 kPa are shown for 1.25% solid-content 3D agarose matrices, yet, matrices with a higher solid content and higher stiffness levels were unused in the analysis pipeline due to a decreased displacement signal-to-noise ratio.

## Conclusion

To summarize, we use magnetic microrheology for the first time to quantify microscale viscoelasticity of several 3D culture matrices [18, 38, 40–43, 58] and stiffness levels, which are relevant to breast-tumor tissue. Specifically, the measurements stiffness (E) levels between 100 Pa and 10 kPa cover the most of breast tumor-stiffness range [9, 44, 59]. Several aspects have been advanced in relation to the previously developed microrheology technique [33], including the microrheometer calibration, heterogeneity quantification, and the pointwise microscale measurements up to Young’s moduli of 10 kPa. Particularly, the heterogeneity quantification using the Bayesian multilevel model contributes to probe-based magnetic rheology by providing insight into different matrix types’ heterogeneity, experimental design, and estimation of measurement errors. These advanced microrheology methods pave the way for enhanced use of 3D cell cultures in breast-cancer research.

## Supporting information

S1 File Supporting information contains all the supporting tables, figures and calculations. (PDF)
