# Supplementary material for: Magnetic microrheometry of tumor-relevant stiffness levels and probabilistic quantification of viscoelasticity differences inside 3D cell culture matrices
Source: PLoS One. 2023 Mar 22;18(3):e0282511. doi: 10.1371/journal.pone.0282511 (PMC10032533; doi:10.1371/journal.pone.0282511)
Supplement: S1 File — (PDF) [file pone.0282511.s001.pdf]

Supporting Information: Magnetic microrheometry of tumor-relevant stiffness levels and probabilistic quantification of viscoelasticity differences inside 3D cell culture matrices

Arttu J. Lehtonen[1,¶], Ossi Arasalo[1,¶], Linda Srbova[1], Maria Heilala[2], and Juho Pokki[1*]

1 Department of Electrical Engineering and Automation, Aalto University, Espoo, Finland
2 Department of Applied Physics, Aalto University, Espoo, Finland

¶, These authors contributed equally to this work.
*Corresponding author, with e-mail: juho.pokki@aalto.fi

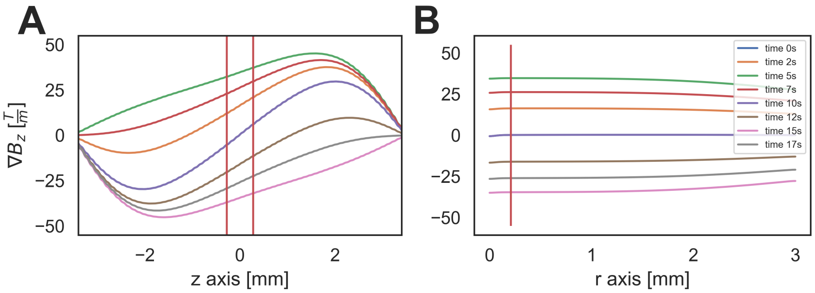

Figure S1: **Comsol simulation of magnetic-field gradients. A-B:** Simulation shows the degree of homogeneity for the magnetic-field gradients within the experimental workspace over the duration of the sinusoidal forces exertion (i.e. 20 s). Horizontal red lines indicate the boundaries of the magnetic hotspot, which is the experimental workspace. The simulation is based on [1] and uses rotational symmetry in cylindrical coordinates.

Materials and methods: data-processing pipeline. To measure microscale viscoelasticity, videos on magnetic and reference sphere displacements are captured. The videos needs to be transformed into the viscoelasticity data. The data-processing pipeline for the purpose is written in Python and operates mostly automatically. Still, user inputs are always required in the step 1 and in the steps 3–4 if the quality of the data is lower. The pipeline works as follows:

1. Magnetic sphere tracking, consisting of automatic tracking with manual initialization.

(a) Manually initialize bounding boxes for magnetic and reference spheres
(b) Tracker binarizes the bounding boxes with Otsu-Binarization
(c) Tracker finds the biggest "blob" for each bounding box and computes the center point
(d) The bounding box is centered based on the computed new center point
(e) Repeat (b–d) over the entire video

2. Track association, involving with matching the IDs over repeated measurements. The same magnetic sphere should have the same ID over the repeats. This step is only necessary if the spheres are not tracked in the same order over the repeats (i.e bounding boxes are not drawn in the same order for each repeat).

   (a) DBSCAN is used to cluster tracked the xy-coordinates
   (b) New IDs for each magnetic sphere based on the cluster assignment

3. Magnetic sphere radius estimation, including an estimate of the magnetic sphere radius.

   (a) The first repeat of a measurement set should have a sweep video through the z-stack
   (b) Estimate the optimal binarization over the video
   (c) Binarize
   (d) Find the best focus by calculating the Laplacian of a bounding box and calculating the variance (for each magnetic sphere separately). The best focus should have the highest variance.
   (e) Estimate the radius by matching the smallest bounding circle over the magnetic sphere at the best focus point
   (f) Estimate manually if not satisfactory

4. Synchronize current output with the sphere displacement. The current input and the video recorder are started separately from different computers so there is an offset.

   (a) Match the synchronization peaks by calculating the cross correlation and finding the maximum.
   (b) If fails, synchronize manually

5. Find the viscoelastic properties from the displacement information

   (a) Subtract reference sphere signal from the magnetic sphere signals
   (b) Fit sinusoidal curve (scipy's dogbox algorithm)
   (c) Filter out poor signals
      i. If reference sphere is too far or right next to the magnetic sphere (i.e. we define that the distance range between a magnetic and a reference sphere is from 50 μm to 250 μm)
      ii. If amplitude is zero
      iii. If the shape of the sinusoidal signal is not symmetric enough (i.e. detrended displacement signal should have similar area for the two half periods)

Materials and methods: the $\frac{|G_{100}|}{|G_{30}|}$ ratio. By using the given formula for absolute shear modulus ($|G|$) in Eq. 4, we can derive an approximation for the $\frac{|G_{100}|}{|G_{30}|}$ ratio, as followed:

$$
\begin{aligned}
|G| &= \frac{\hat{F}_{\text{volumetric}} V_{\text{sphere}}}{3\pi D_{\text{sphere}} \hat{p}_{\text{sphere}}}, \quad \text{where } V_{\text{sphere}} = \frac{4}{3}\pi (D_{\text{sphere}})^3 \\
|G| &= \frac{\hat{F}_{\text{volumetric}} \frac{4}{3}\pi (D_{\text{sphere}})^3}{3\pi D_{\text{sphere}} \hat{p}_{\text{sphere}}} \\
|G| &= \frac{\hat{F}_{\text{volumetric}} \frac{4}{3} (D_{\text{sphere}})^2}{3 \hat{p}_{\text{sphere}}} = \frac{4}{9} \frac{\hat{F}_{\text{volumetric}} (D_{\text{sphere}})^2}{\hat{p}_{\text{sphere}}}
\end{aligned}
$$

This information is plugged into the calculation of the ratio:

$$
\frac{|G_{100}|}{|G_{30}|} = \frac{\frac{\hat{F}_{\text{volumetric},100} (D_{\text{sphere},100})^2}{\hat{p}_{\text{sphere},100}}}{\frac{\hat{F}_{\text{volumetric},30} (D_{\text{sphere},30})^2}{\hat{p}_{\text{sphere},30}}}
$$

- The ratio between the volumetric force constants: $\frac{\hat{F}_{\text{volumetric},100}}{\hat{F}_{\text{volumetric},30}} \approx 0.75$
- We want to explore the $|G|$ values at the resolution limit range of the microrheometer: $\hat{p}_{\text{sphere},100} \approx \hat{p}_{\text{sphere},30} < 100$ nm

$$
\begin{aligned}
\frac{|G_{100}|}{|G_{30}|} &= 0.75 \frac{(D_{\text{sphere},100})^2}{(D_{\text{sphere},30})^2} = 0.75 \frac{(100\mu m)^2}{(30\mu m)^2} \\
\frac{|G_{100}|}{|G_{30}|} &= 0.75 \frac{(100\mu m)^2}{(30\mu m)^2} = 8.367... \approx 8
\end{aligned}
$$

Therefore, we conclude that the 100 μm magnetic spheres are expected to measure approximately 8 times larger stiffness levels than the 30 μm magnetic spheres.

Materials and methods: model comparison. As described in the methods and materials section, the heterogeneity model (Model 1, Fig. 2) was compared against four other variants of the model (Models 2–5, Figs S1–4). The results are summarized in Table S1, where a higher expected log pointwise predictive density (ELPD) value denotes a better predictive performance. The Model 1 has been chosen as the primary model due to its ability to capture interpretable experimental design and has the second-highest ELPD for $|G|$ estimation. The Model 2 has the highest ELDP, which is however within the standard errors for the Models 1–2, and the heterogeneity quantification by the Model 2 is uninterpretable as described in Materials and Methods. The choice of the Models 1–2 for estimating $|G|$ has a slight improving impact on the model performance based on the ELPD, while the most impacting aspect on the performance is to have a unique noise for each magnetic sphere. The unique noise term is needed, because particle tracking and radius estimation are done separately for each magnetic sphere producing different levels of noise. For estimating ϕ,

adding hierarchy to the model has no noticeable effect on the performance based on this ELPD analysis (i.e. Model 1 has the lowest ELPD, yet, all the models have a close ELPD that are within standard errors of the Model 3). To summarize, the choice of the Model 1 was mainly done based on the performance to estimate the $|G|$ and the interpretability (i.e. Models 2–5 are partially uninterpretable for the purpose).

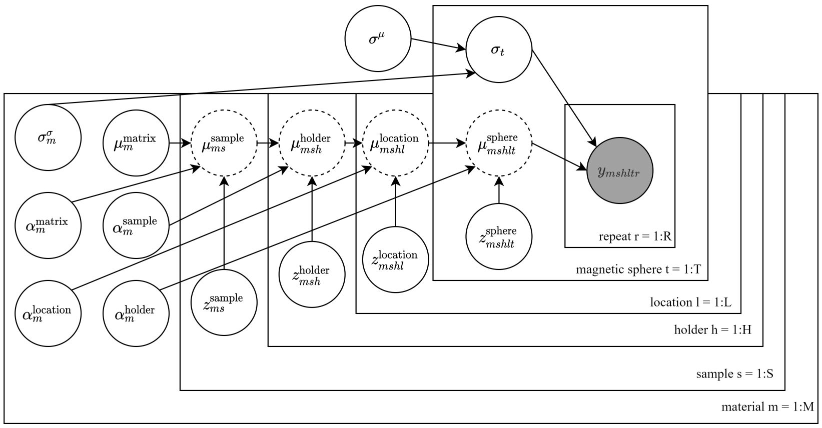

Figure S2: **Model 2 definition.** The model includes an additional location level effect. The priors are same as in the Model 1.

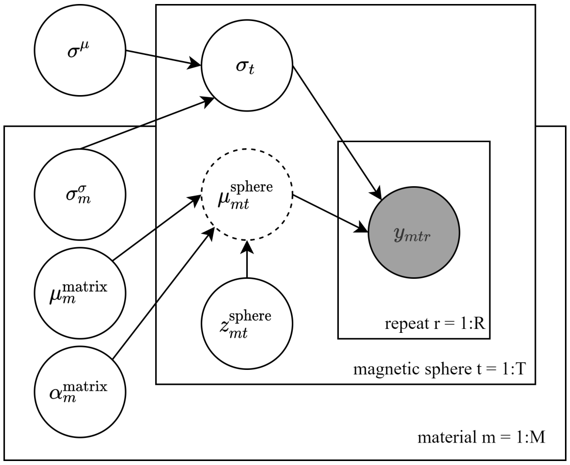

Figure S3: **Model 3 definition.** The model has only a single level of hierarchy.

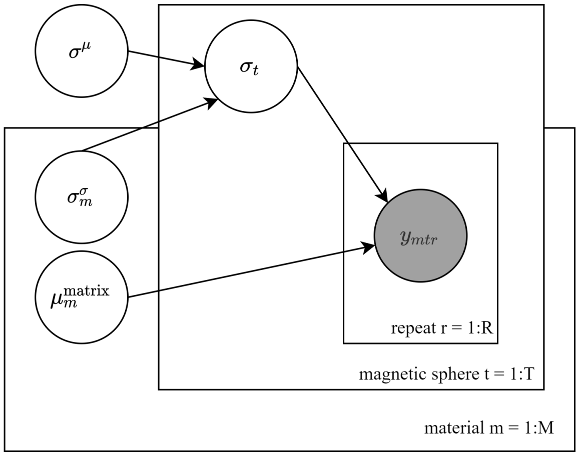

Figure S4: **Model 4 definition.** Only a single parameter for each matrix-type material defines the viscoelastic property for a single magnetic sphere.

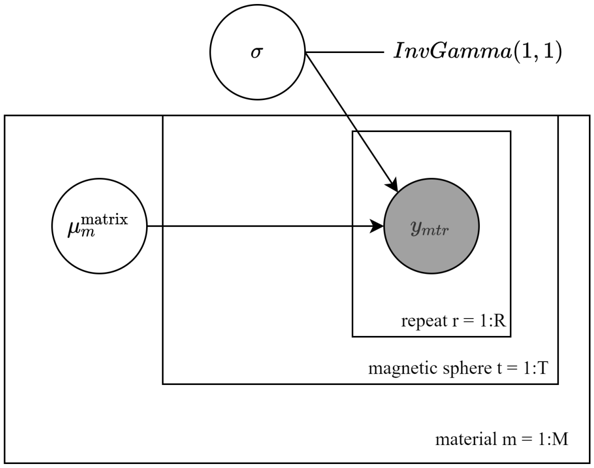

Figure S5: **Model 5 definition.** Only a single parameter for each matrix-type material defines the viscoelastic property and the measurement noise for a single magnetic sphere.

Table S1: **Model comparisons based on ELPD and standard error.** A higher ELPD value indicates better model performance. The choice of the model is considered more important for estimating $|G|$. Relatively little gain is achieved between the models when estimating ϕ.

ELPD	standard error	Model	Property
-153.505372	17.654360	2	G
-162.652745	16.678239	1	G
-166.733471	16.629869	3	G
-169.539086	16.934008	4	G
-284.080725	58.914523	5	G
ELPD	standard error	Model	Property
-166.883241	14.163177	3	ϕ
-171.043780	14.657215	5	ϕ
-172.631493	15.575221	4	ϕ
-175.044546	15.681705	2	ϕ
-175.160629	15.562709	1	ϕ

Materials and methods: effect of the spheres

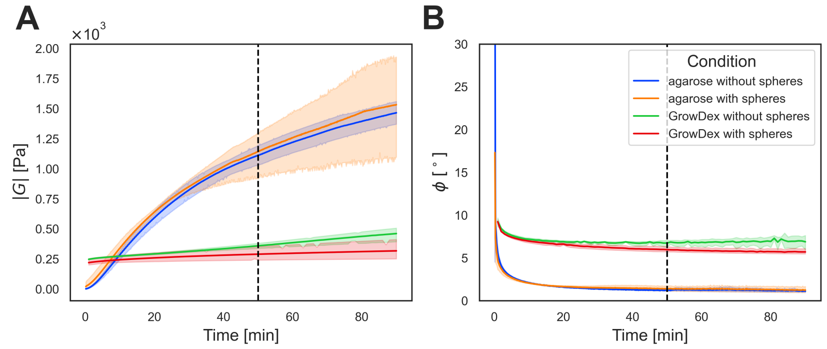

Figure S6: **Parallel-plate rheometry of viscoelastic properties of 3D-culture matrices with the spheres, and without the spheres.** **A**–**B**: No significant differences in shear modulus ($|G|$) and phase angle (ϕ) are found between the matrices without the spheres, and with the magnetic/reference spheres. The data is for agarose matrices (a 0.9% concentration) and GrowDex matrices (a 1.25% concentration). Comparable viscoelasticity measurements have been previously established for accurate, cell-scale bulk properties of collagen matrices [2]. Because magnetic spheres can measure both the GrowDex and collagen matrices properties, it is reasonable to assume that GrowDex–collagen matrices are similarly measurable.

Materials and methods: calibration. For calibration, the initial estimation method is based on calculating the volumetric force constant as a mean value of each individual magnetic sphere's volumetric force, showing a method-related scatter of individual sphere results (Table S2). Here, instead, we use a more accurate, enhanced estimation method. Our approach is directly based on the definition of the force from Stokes' law and a linear regression model (Eq. 6–9). The data in Table S2 follows the linear model, as expected, and the Bayesian R^2 [3] (goodness of fit) is reasonable for all

cases. Notably, the R^2 values are 0.88 for the 30 μm spheres (i_{grad}=1.25 A) and 0.82 for the 100 μm spheres (i_{grad}=1.25 A). NB: we use the current (i_{grad}=1.25 A) for all further experiments and analyses. Based on the low errors shown in Table S2, this enhanced estimation improves reliability.

Table S2: **Comparison of the enhanced (enh.) and initial (init.) estimates of $\hat{F}_{\textbf{volumetric}}$ for the 30 μm and the 100 μm spheres.** The error is the percentual ratio between standard deviation and mean.

i_{grad} [A]	mean $[\frac{N}{m^3}]$		σ $[\frac{N}{m^3}]$		error [%]	
	enh.	init.	enh.	init.	enh.	init.
0.65 (30 μm)	133566	151514	10119	47201	7.6	31.2
1.00 (30 μm)	199904	204502	10982	46638	5.5	22.8
1.25 (30 μm)	259266	270298	9259	45142	3.6	16.7
1.25 (100 μm)	195413	205337	6719	38598	3.4	18.8

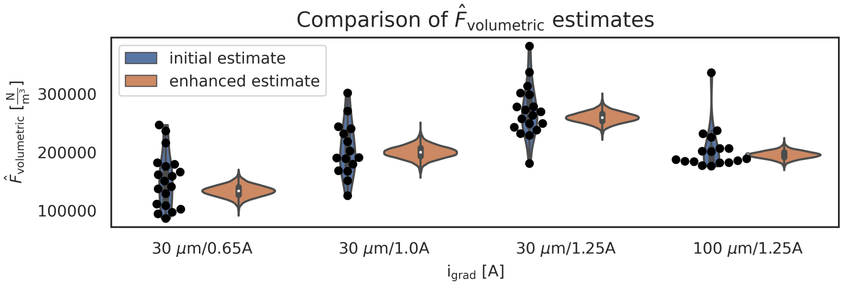

Figure S7: **Graph of calibration values based on the initial and enhanced estimates of $\hat{F}_{\textbf{volumetric}}$.** The graph compares the $\hat{F}_{\text{volumetric}}$ constants by the initial estimate (calculated separately for each sphere) and the enhanced estimate (calculated using Stokes' law). Both of the estimates provide similar mean values. Importantly, the graph illustrates how the enhanced estimate has consistently lower uncertainty than the initial estimate. The estimates show that the $\hat{F}_{\text{volumetric}}$ values are increasing linearly as a function of the current i_{grad} for the 30 μm spheres. Each dataset is either for the 30 μm or the 100 μm spheres.

Table S3: **Number of datapoints from all experiments that have passed the post-processing analysis pipeline**

Matrix type	Microrheology: number of magnetic spheres	Parallel-plate rheometry: number of samples	Heterogeneity estimation: samples / holders/ locations / magnetic spheres / repeats
Calibration (the 30 μm spheres)	14	N/A	N/A
Calibration (the 100 μm spheres)	12	N/A	N/A
Agarose 0.5%	71	3	3 / 6 / 24 / 71 / 1-3
Agarose 0.9%	19	4	N/A
Agarose 1.0%	33	4	N/A
Agarose 1.1%	12	3	N/A
Agarose 1.25%	18	5	N/A
Agarose 1.35%	16	2	N/A
GrowDex–collagen 0.45%/2.0 mg/mL	44	3	3 / 6 / 14 / 44 / 1-3
GrowDex 1.25%	29	3	3 / 5 / 23 / 29 / 1-3
Fibrin 30 mg/mL	47	3	3 / 8 / 24 / 47 / 1-3

Results: heterogeneity

Table S4: **Pairwise comparisons of the heterogeneity in viscoelasticity for the different matrix types.** Posterior distributions of α_m^{holder} have been compared to provide a probability value quantifying pairwise differences. Only non-zero probabilities are reported.

Pairwise heterogeneity difference	Probability
$\|G\|$	
Pr(Agarose > Fibrin)	0.1964
Pr(Agarose > GrowDex–collagen)	0.0495
Pr(Agarose > GrowDex)	0.0056
Pr(Fibrin > Agarose)	0.8036
Pr(Fibrin > GrowDex–collagen)	0.1941
Pr(Fibrin > GrowDex)	0.0698
Pr(GrowDex–collagen > Agarose)	0.9505
Pr(GrowDex–collagen > Fibrin)	0.8059
Pr(GrowDex–collagen > GrowDex)	0.2657
Pr(GrowDex > Agarose)	0.9944
Pr(GrowDex > Fibrin)	0.9303
Pr(GrowDex > GrowDex–collagen)	0.7342
ϕ	
Pr(Agarose > Fibrin)	0.9591
Pr(Agarose > GrowDex)	0.0726
Pr(Fibrin > Agarose)	0.0409
Pr(Fibrin > GrowDex)	0.0147
Pr(GrowDex-collagen > Agarose)	0.9999
Pr(GrowDex–collagen > Fibrin)	0.9999
Pr(GrowDex–collagen > GrowDex)	0.9951
Pr(GrowDex > Agarose)	0.9274
Pr(GrowDex > Fibrin)	0.9852
Pr(GrowDex > GrowDex–collagen)	0.0049

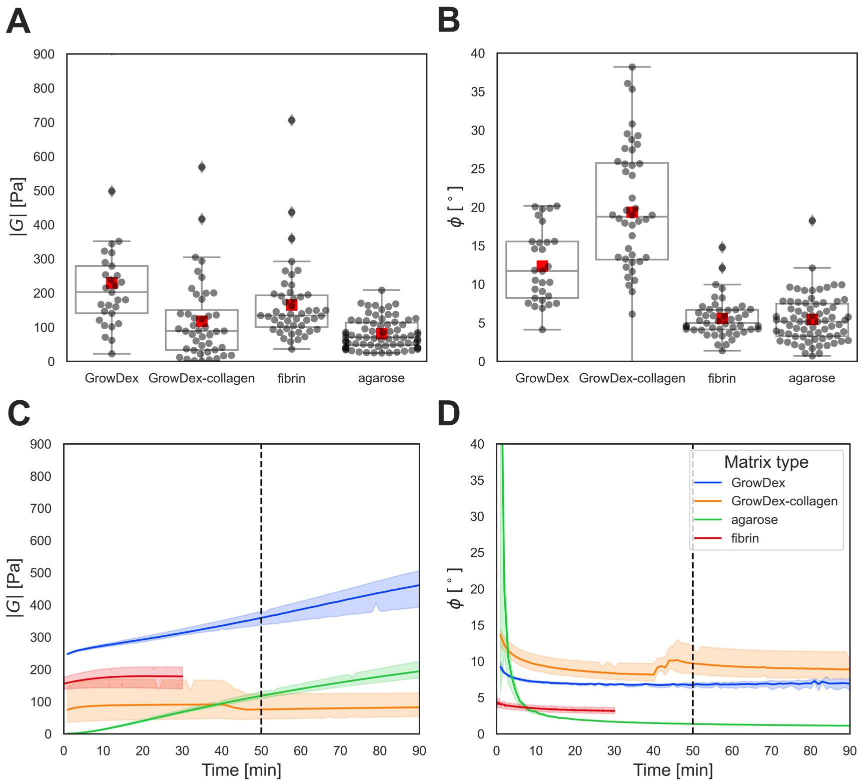

Figure S8: **Viscoelasticity of the matrix types based on microrheometry and parallel-plate rheometry.** **A–B**: Absolute shear modulus ($|G|$) and phase angle (ϕ) results of the microrheometry measurements for the used matrices types are shown. The compositions of the matrices are as followed: GrowDex has a concentration of 1.25 %; GrowDex–collagen has a GrowDex's concentration of 0.45 % and a collagen concentration of 2.0 mg/mL; fibrin has a concentration of 30 mg/mL; and agarose has a concentration of 0.5 %. For both plots, the dark grey spheres show values measured by individual magnetic spheres, while the red squares are the mean values of the spheres. Standardly, the plots' box and whiskers indicate the lower/upper extremes, the 25 % and the 75 % percentiles, and the median. **C–D**: Absolute shear modulus ($|G|$) and phase angle (ϕ) results of the parallel-plate rheometry measurements for the used matrices types are shown. Black vertical dashed line indicates the timepoint when the microrheological measurements have been started for all matrix types apart from the fibrin matrix. Fibrin-matrix measurements were started after 30 min. The curves with darker colors indicate the mean values of the data, while the lighter shaded-color regions indicate the lower 5 % and the upper 95 % percentiles for the data.

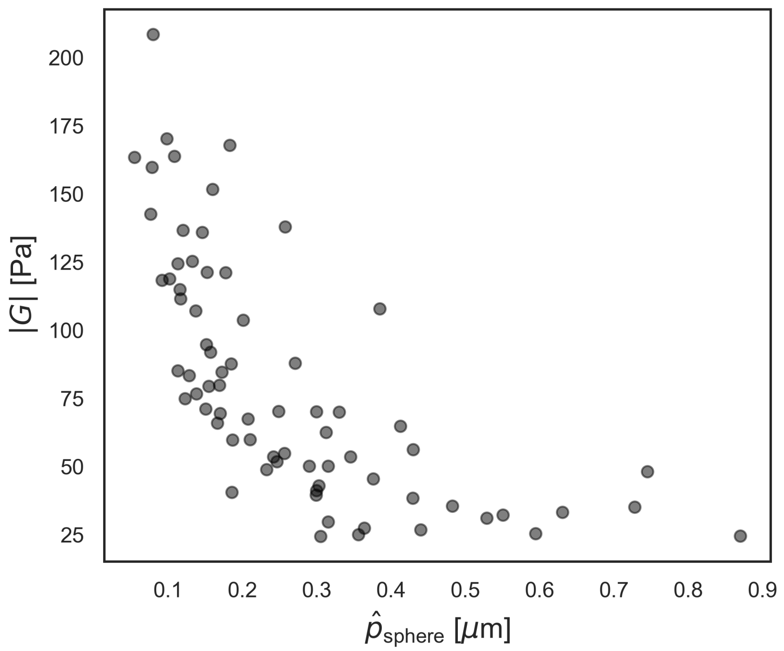

Figure S9: **Absolute shear modulus in relation to the displacement signal amplitude.** Absolute shear modulus ($|G|$) has a multiplicative inverse dependence on the amplitude of the displacement signal ($\hat{p}_{\text{sphere}}$). An increased uncertainty of $|G|$ at low displacement amplitude values is shown (i.e. smaller differences in the displacement amplitude cause larger differences in $|G|$ at lower displacement amplitudes compared to larger displacement amplitudes).

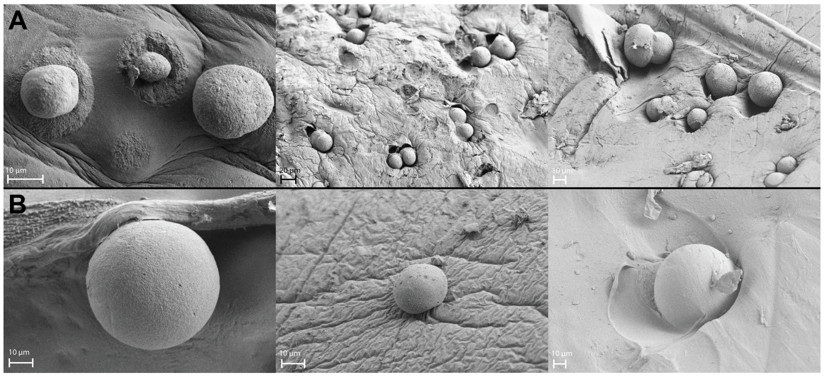

Figure S10: **SEM micrographs of agarose matrices with the 30μm and the 100μm spheres.** SEM micrographs illustrate the occasional deviation of (**A:**) the 30μm and (**B:**) the 100μm magnetic spheres from a perfect spherical shape, which is expected to be an error source in our microrheometry methods and detectable in the calibration.

References

[1] Granum P, Madsen ML, McKenna JTK, Hodgkinson DL, Fajans J. Efficient calculations of magnetic fields of solenoids for simulations. Nuclear Instruments and Methods in Physics Research Section A: Accelerators, Spectrometers, Detectors and Associated Equipment. 2022;1034:166706.

[2] Pokki J, Zisi I, Schulman E, Indana D, Chaudhuri O. Magnetic probe-based microrheology reveals local softening and stiffening of 3D collagen matrices by fibroblasts. Biomedical microdevices. 2021;23(2):1–14.

[3] Gelman A, Goodrich B, Gabry J, Vehtari A. R-squared for Bayesian regression models. The American Statistician. 2019;.
